# Publisher Correction: Infused-liquid-switchable porous nanofibrous membranes for multiphase liquid separation

**DOI:** 10.1038/s41467-017-02010-4

**Published:** 2017-11-30

**Authors:** Yang Wang, Jiancheng Di, Li Wang, Xu Li, Ning Wang, Baixian Wang, Ye Tian, Lei Jiang, Jihong Yu

**Affiliations:** 1 State Key Laboratory of Inorganic Synthesis and Preparative Chemistry, College of Chemistry, Jilin University, Changchun, 130012 China; 2 Key Laboratory of Bio-inspired Materials and Interfacial Science, Technical Institute of Physics and Chemistry, Chinese Academy of Sciences, Beijing, 100190 China; 3 Beijing National Laboratory for Molecular Sciences (BNLMS), Key Laboratory of Green Printing, Institute of Chemistry, Chinese Academy of Sciences, Beijing, 100190 China; 4 University of Chinese, Academy of Sciences, Beijing, 100049 China; 5 International Center of Future Science, Jilin University, Changchun, 130012 China

*Nature Communications* **8**:575 doi:10.1038/s41467-017-00474-y; Article published online: 18 September 2017

In Table 1 of this Article, the third row in the furthest right column contains a typographical error and should read ‘NH’, rather than ‘NM’, corresponding to *n*-hexane as the infused liquid.

