# Supplementary material for: Infused-liquid-switchable porous nanofibrous membranes for multiphase liquid separation
Source: Nat Commun. 2017 Sep 18;8:575. doi: 10.1038/s41467-017-00474-y (PMC5603539; doi:10.1038/s41467-017-00474-y)
Supplement: Supplementary file 1 — Supplementary Information [file 41467_2017_474_MOESM1_ESM.pdf]

Description of Supplementary Files

File Name: Supplementary Information
Description: Supplementary Figures, Supplementary Tables, Supplementary Notes and Supplementary References

File Name: Peer Review File

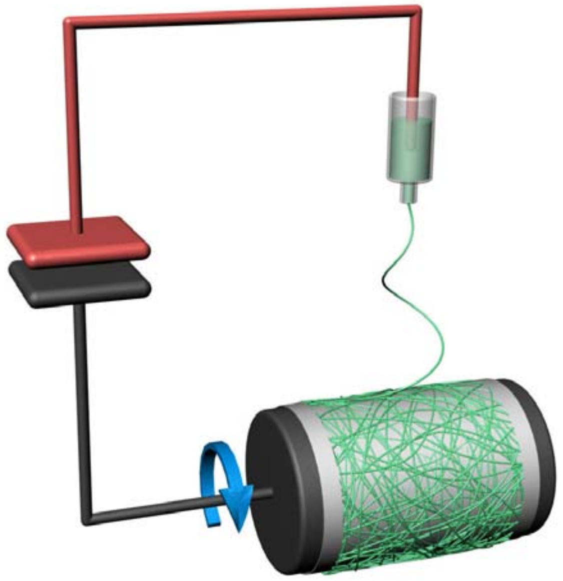

Supplementary Figure 1. Schematic illustration of the fabrication of SiO_2–TiO_2 composite porous nanofibrous membranes (STPNMs) by electrospinning.

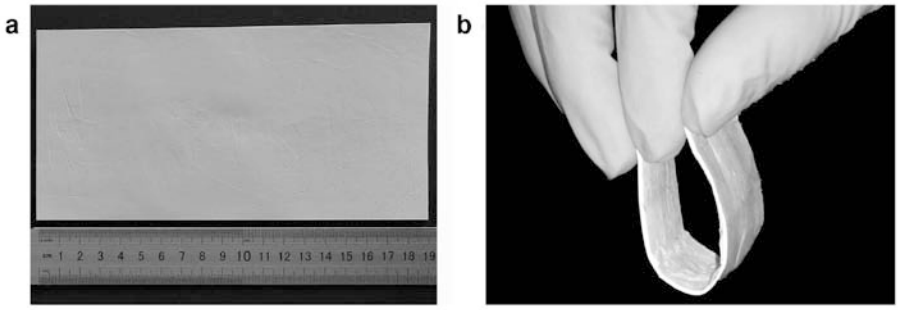

Supplementary Figure 2. Large-area optical images of STPNMs, showing excellent flexibility.

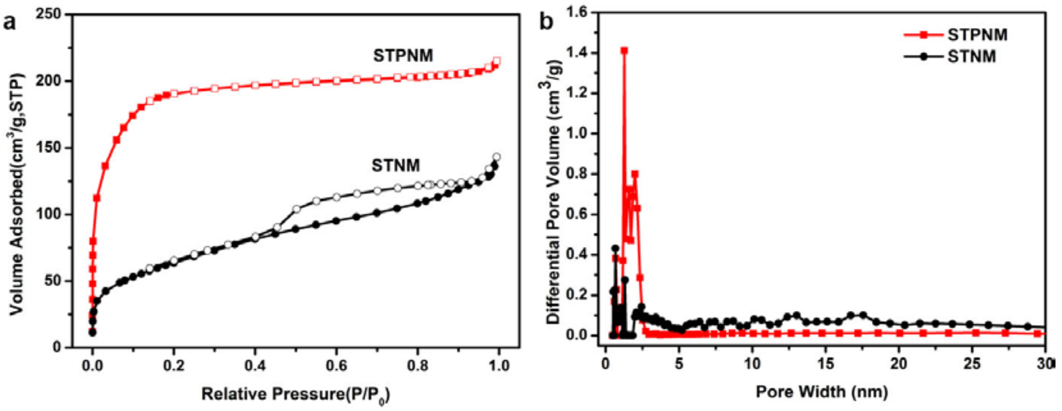

Supplementary Figure 3. (a) Nitrogen adsorption–desorption isotherms and (b) the corresponding pore size of STPNMs and STNMs.

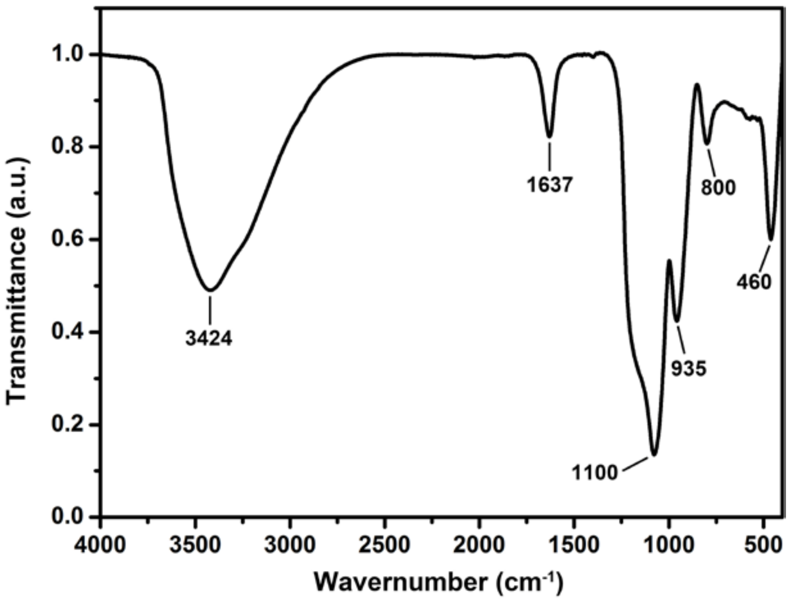

Supplementary Figure 4. FT-IR spectrum of STPNMs. The appearance of absorption bands around 1100, 800, 460 cm^{-1} correspond to Si–O–Si bond[6]. The position of the Ti–O–Si band is around 935 cm^{-1} and the peak at 400-600 cm^{-1} reveals the presence of the Ti–O–Ti bond[7]. The band at 1637 cm^{-1} is attributed to bending of water molecules. The wide peak band at 3424 cm^{-1} indicates the presence of terminal hydroxyl groups[8,9].

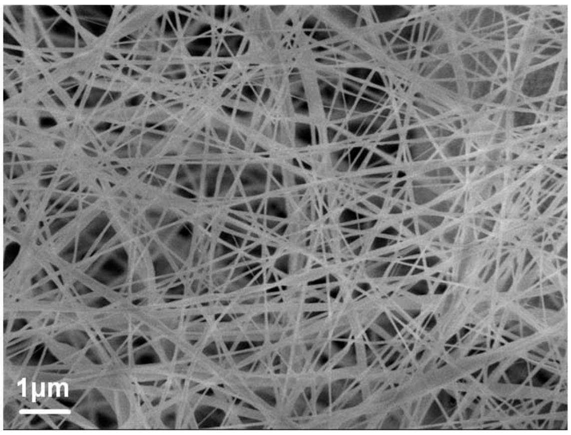

Supplementary Figure 5. SEM image of STNMs, showing the entangled uniform fibers with average diameter of about 200 nm.

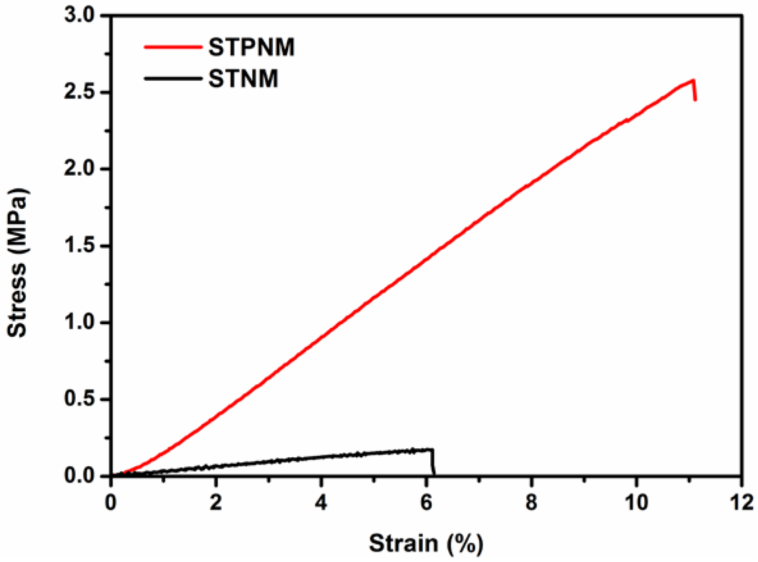

Supplementary Figure 6. The stress-strain curves of STPNMs and STNMs. The derived Young's modulus and stress of break of STPNMs are 25.95 ± 1.9 and 2.93 ± 0.08 MPa, respectively, which are much larger than those of STNMs (2.55 ± 0.11 and 0.183 ± 0.006 MPa). The s.d. is obtained from the test results of at least five replicates.

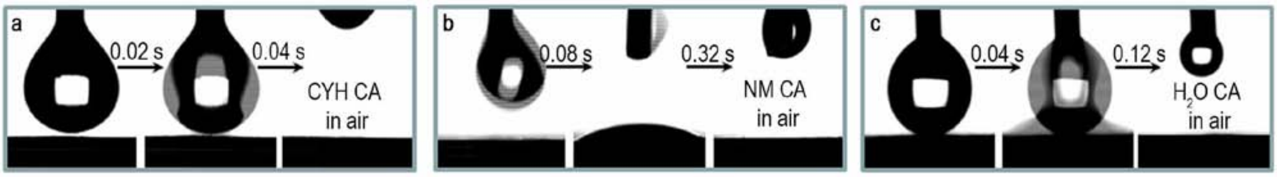

Supplement Figure 7. Wettability of STPNMs. Contact angles (CAs) of CYH, NM and H_2O droplets on STPNMs in air, respectively. The droplets spread out and infiltrate into the membrane rapidly, and the contact angle is almost 0°, showing superamphiphilicity.

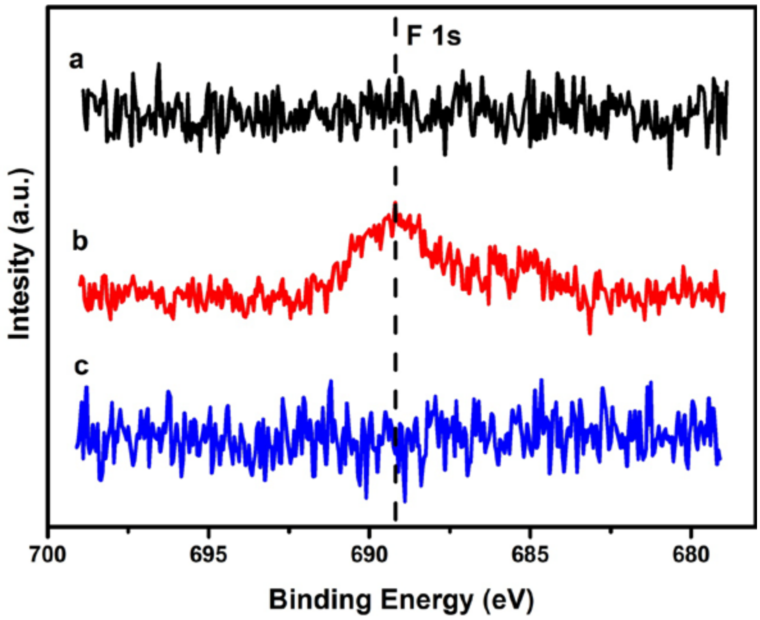

Supplement Figure 8. XPS spectra of STPNM for F element. (**a**) Original STPNM, (**b**) Fluorinert FC-43 infused STPNM, (**c**) STPNM which is first infused by FC-43 and then washed by water.

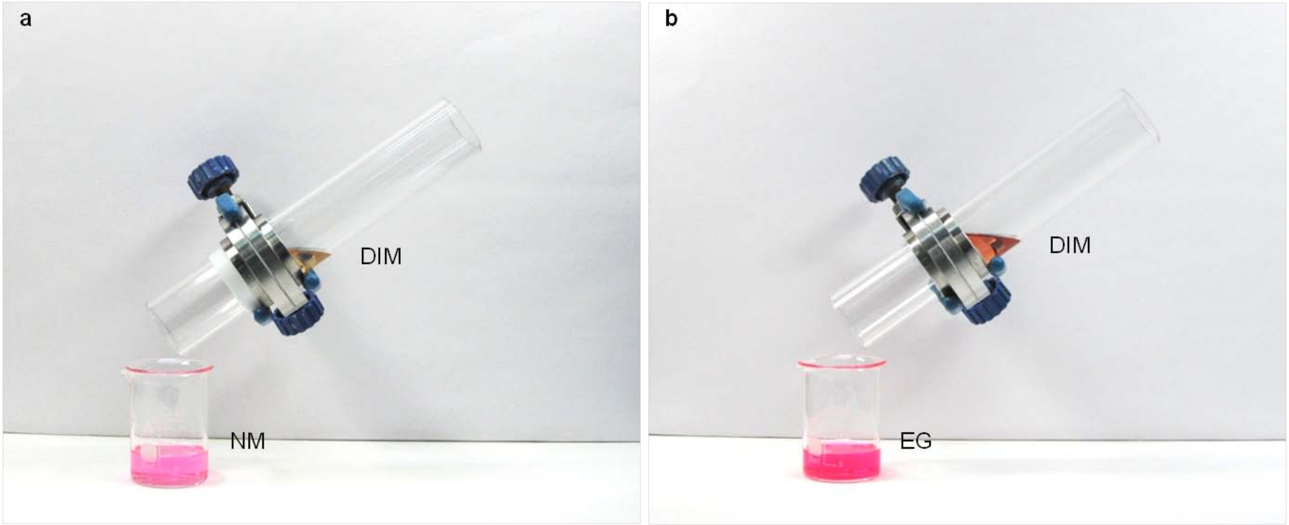

Supplement Figure 9. The separation of NM/DIM and EG/DIM. (**a**) The separation of NM and DIM with NM-LII. (**b**) The separation of EG and DIM with EG-LII.

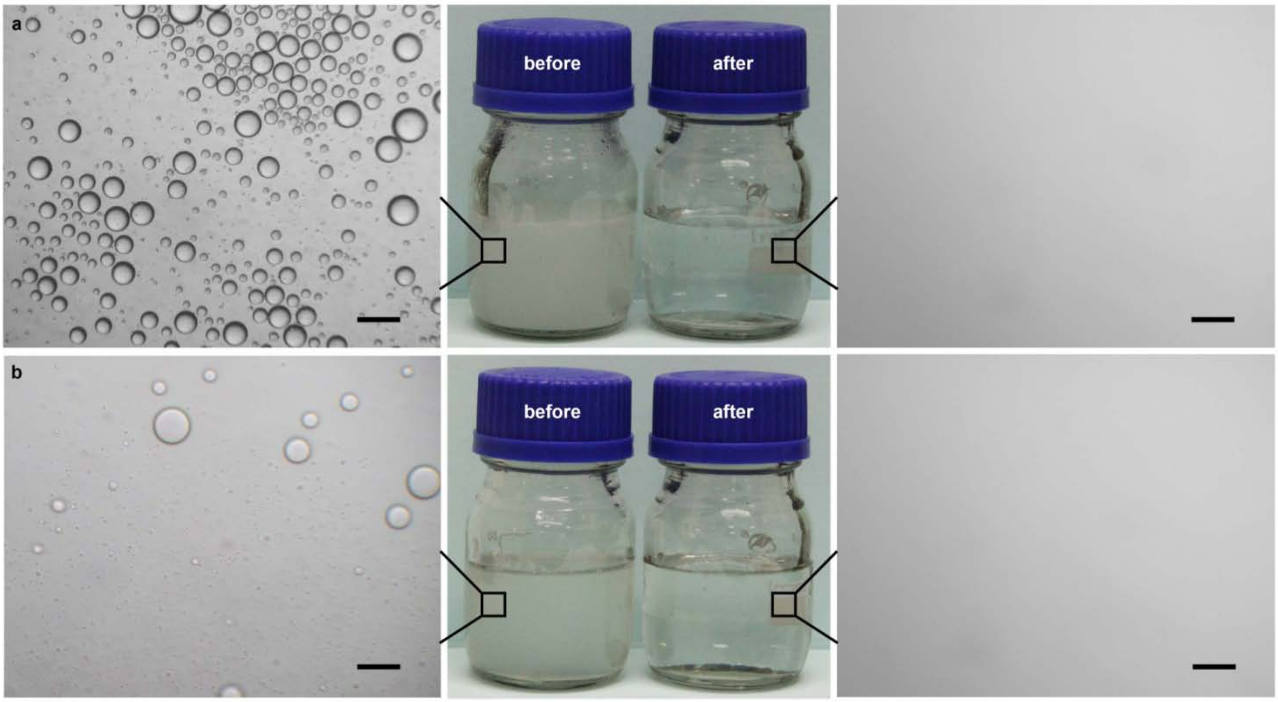

Supplement Figure 10. The emulsion separation of STPNMs. (**a**) Oil-in-water emulsion, cyclohexane/water, stabilized by surfactant CTAB. (**b**) Oil-in-oil emulsion, cyclohexane/formamide, stabilized by surfactant Pluronic F-127. Scale bar, 100 μm. The emulsions are prepared by mixing liquids with volume ratio of 1:100, and then 4 mg/mL of surfactant is added under high stirring. 60 mL emulsion can be separated within 10 minutes.

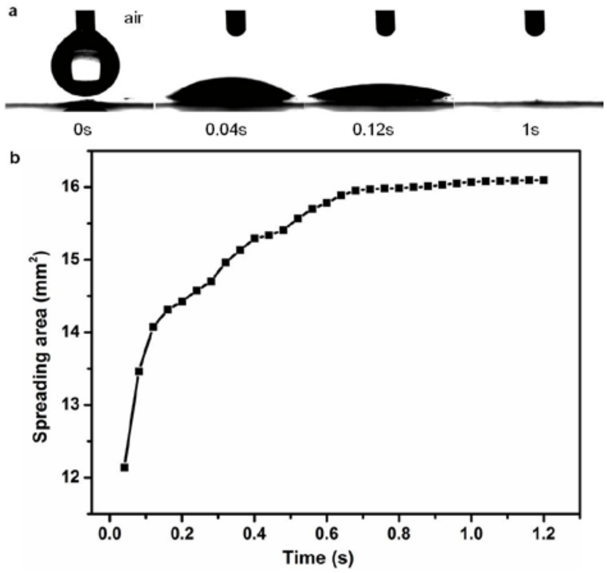

Supplementary Figure 11. Time-scale of the water substitution into the nitromethane infiltrated membranes. (**a**) Water drop (3 μL) spreads out quickly once contact with the nitromethane infused STPNMs and infiltrates into the membrane within 1s. (**b**) Variation of the spreading area of a water drop (3 μL) on the nitromethane infused STPNMs.

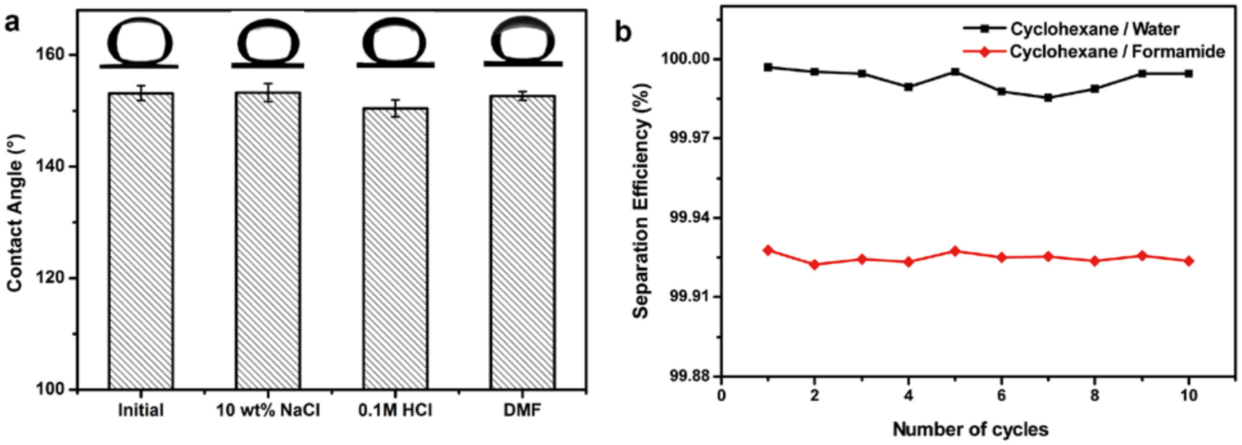

Supplementary Figure 12. Chemical stability and recyclability of STPNMs. (**a**) The underwater oil CAs (CCl_4) of the PFMs after being immersed in NaCl (10 wt %), HCl (0.1 M) aqueous solutions and DMF for 20 days, respectively. It can be seen that the PFMs retain their underwater superlyophobic property and no obvious change of CA is observed after immersion. The error bars represent the s.d. obtained from the test results of at least five replicates. (**b**) Recyclability and the separation efficiency of PFMs. The separation of immiscible liquids, cyclohexane/water and cyclohexane/formamide, for ten times is introduced to test the recyclability of the PFMs. After each separation process, the membrane is treated by heating at 200 ^{o}C for 1 h to remove the adsorbed liquid and then reused. The separation efficiency is above 99.9% and there is no obvious attenuation after ten cycles. The error bars represent the s.d. obtained from the test results of at least five replicates.

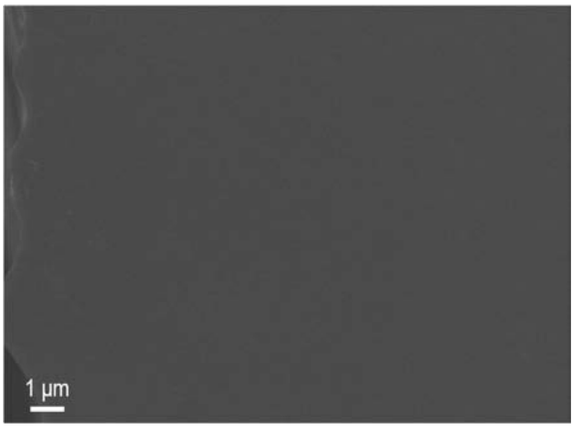

Supplementary Figure 13. SEM image of the flat SiO_2-TiO_2 composite membrane.

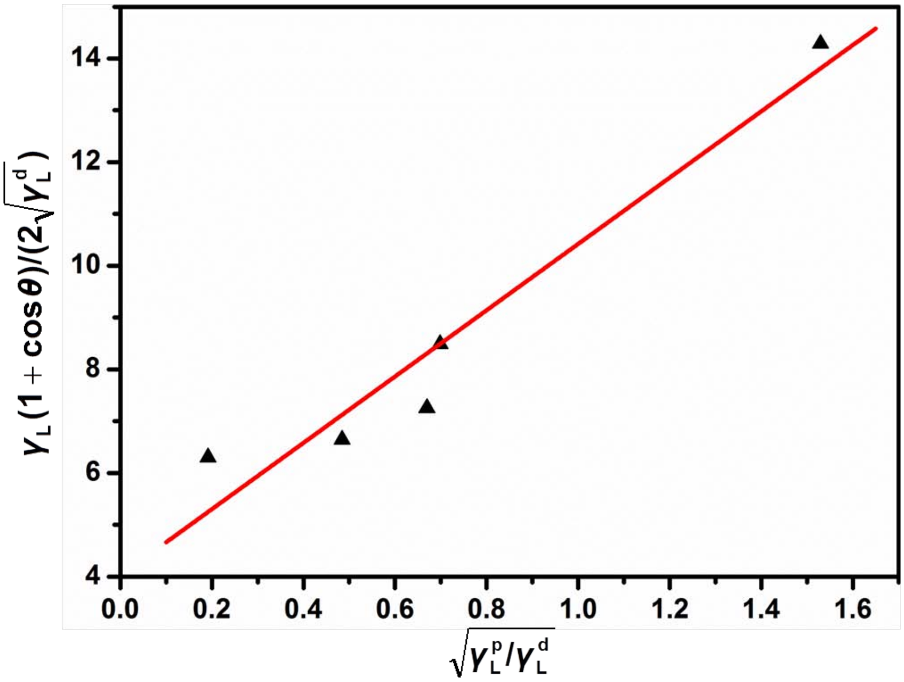

Supplementary Figure 14. Fitted curve of surface tension of STPNMs by OWRK method. Five liquids are used to increase accuracy of the results: water, ethylene glycol, *N*, *N'*-dimethylformamide, nitromethane and diiodomethane. The estimated results are: γ_S = 57.11 mN m^{-1}, γ_S^d = 18.18 mN m^{-1}, γ_S^p = 40.93 mN m^{-1}.

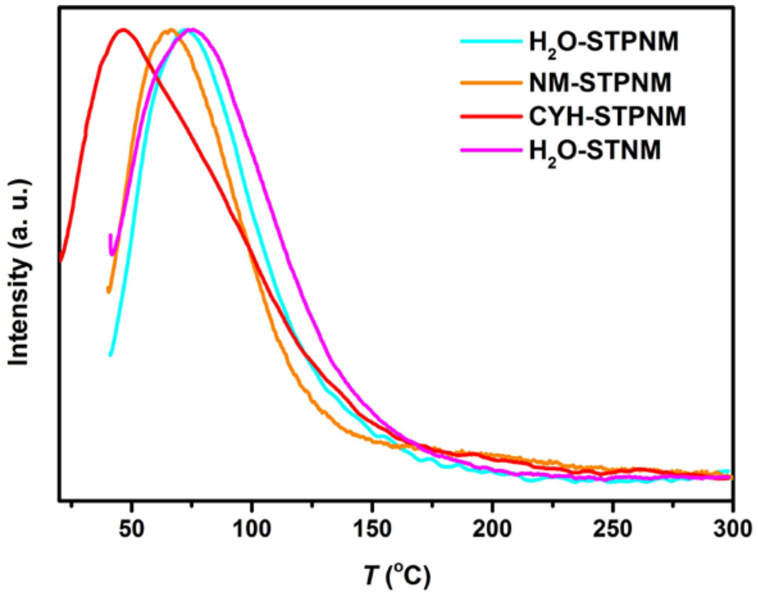

Supplementary Figure 15. Normalized liquid-TPD profiles of STPNMs and STNMs with temperature ramping rate of 5 °C min^{-1}. The temperature of desorption peaks of water, NM and CYH on STPNMs appear at 72.4 ± 0.3 °C, 66.3± 0.5 °C and 46.9 ± 0.4 °C, respectively, and water on STNMs at 73.8± 0.5 °C. The s.d. is obtained from the test results of at least three replicates.

Supplementary Table 1. Components of surface energy for various liquids.

Liquid	Abv.	SE (γ) mJ m^{-2}	DSE (γ^d) mJ m^{-2}	PSE (γ^p) mJ m^{-2}
water	H_2O	72.8	21.8	51[1]
formamide	FM	58	39	19[1]
diiodomethane	DIM	50.8	49	1.8[2]
ethylene glycol	EG	48.8	32.8	16[3]
dimethylsulfoxide	DMSO	44	36	8[1]
nitromethane	NM	36.8	29.8	7[4]
N, *N'*-dimethylformamide	DMF	36.5	25.2	11.3[5]
ethane dichloride	ED	33.3	30.8	2.5[4]
toluene	TL	28.4	26.1	2.3[4]
tetrachloromethane	CCl_4	27	26.7	0.3[4]
cyclohexane	CYH	25.24	25.24	0[1]
kerosene	KS	25.22	25.22	0
petroleum ether	PE	20.25	20.25	0
n-hexane	NH	18.4	18.4	0[1]
Fluorinert FC-43		16.4	16.4	0

Note: SE: surface energy, DSE: dispersive component of surface energy, PSE: polar component of surface energy. SE measurements for kerosene, petroleum ether and Fluorinert FC-43 were performed by the pendant drop method at 20 ^oC and their PSE is approximated as 0.

Supplementary Table 2. Contact angles of various liquids on flat SiO_2–TiO_2 composite membranes.

Liquid	Contact angle (^{o})
water	33.6 ± 4.1
diiodomethane	42.6 ± 2.8
ethylene glycol	7.0 ± 0.5
nitromethane	13.7 ± 0.8
N, *N’*-dimethylformamide	5.6 ± 0.5
cyclohexane	< 5

Supplementary Table 3. Comparison of the governing relationships with experimental observations for various STPNM-liquid-A-liquid-B combinations.

Liquid A	Liquid B	R	ΔE mJ m^{-2}	ΔE^p mJ m^{-2}	*Stable Film?* SE	PSE	Exp.
NM	H_2O	2	13.77	27.36	Y	Y	Y
H_2O	NM	2	-13.77	-27.36	N	N	N
CYH	H_2O	2	23.43	33.96	Y	Y	Y
H_2O	CYH	2	-23.43	-33.96	N	N	N
CYH	NM	2	9.66	6.6	Y	Y	Y
NM	CYH	2	-9.66	-6.6	N	N	N
DIM	EG	2	24.09	14.91	Y	Y	Y
EG	DIM	2	-24.09	-14.91	N	N	N
DIM	NM	2	10.72	5.75	Y	Y	Y
NM	DIM	2	-10.72	-5.75	N	N	N

Note: "Y" indicates that liquid B forms a stable film, and does not get displaced by liquid A; whereas "N" indicates that Liquid B is displaced by liquid A. θ_A and θ_B are estimated from the measured static contact angles on flat substrates from at least three individual measurements (see supplementary Table 2). *R* represents the roughness factor of the substrate, and is estimated as 2[11]. γ_A, γ_A^p and γ_B, γ_B^p represent the SEs and PSEs of Liquid A and B, respectively (see supplementary Table 1).

Supplementary Note 1: Theoretical model based on the minimization of a system's free energy

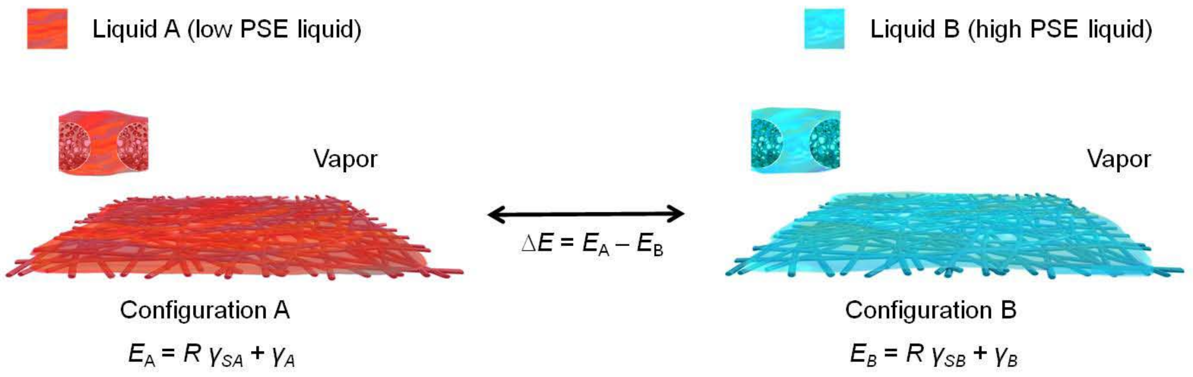

We introduce the theoretical model in terms of the minimization of a system's free energy[10,11] to determine whether a solid will be wetted preferentially by high PSE liquid or low PSE liquid. Configuration A and B refer to the states where the solid is completely wetted by low (E_A) and high (E_B) PSE liquid, respectively.

To find the condition that Configuration A is always at a higher energy state than Configuration B, we have $E_A - E_B > 0$, which can be further expressed as,

$$\triangle E = E_A - E_B = R(\gamma_{SA} - \gamma_{SB}) + \gamma_A - \gamma_B > 0 \quad (1)$$

In particular, Supplementary Equation 1 is reduced to measurable quantities with the use of the Young equation (see, for example, Joanna Aizenberg *et al., Nature*, 2015, 519, 70), where we have,

$$\triangle E = R(\gamma_B \cos\theta_B - \gamma_A \cos\theta_A) + \gamma_A - \gamma_B > 0 \quad (2)$$

Where γ_A and γ_B are the surface tensions for the liquid to be repelled and the infused liquid, respectively, γ_{SB} is the interfacial tension at the solid–liquid interface, θ_A and θ_B are the equilibrium CAs of the repelled liquid and the infused liquid on a flat solid surface, respectively. R represents the roughness factor of the solid, which is defined as the ratio between the actual and projected areas of the surface.

Satisfying Supplementary Equation 2 will ensure a stable LII formation. In contrast, when Supplementary Equation 2 is not satisfied, liquid B will be displaced by liquid A. To verify this theoretical model, we explored a number of different solid/liquid-A/liquid-B combinations and compared these results with the governing relationships. We show that these relationships agree favorably with all of the experimental conditions (see supplementary Table 3).

Furthermore, we replace the energy argument of total SE in Supplementary Equation 1 with PSE to investigate the influence of the PSE of liquid on the stability of liquid-LII and we have $E_A^p - E_B^p > 0$, which can be further expressed as,

$$\triangle E^p = E_A^p - E_B^p = R(\gamma_B^p \cos\theta_B - \gamma_A^p \cos\theta_A) + \gamma_A^p - \gamma_B^p > 0 \quad (3)$$

Where γ_A^p and γ_B^p are the PSEs for the liquid to be repelled and the infused liquid, respectively.

Supplementary Note 2: The OWRK (Owen, Wendt, Rabel and Kaelble) method

For interface of solid surface and liquid, work of adhesion can be expressed as follows:

$$W_a = \gamma_S + \gamma_L - \gamma_{SL} \tag{4}$$

The adhesion can divided into polar and dispersion parts. As proposed by Fowkes, the polar and dispersive interfacial attractions can be treated independently, and the polar-dispersive interactions can be neglected.

$$W_a = W_a^d + W_a^p = 2(\sqrt{\gamma_S^d \gamma_L^d} + \sqrt{\gamma_S^p \gamma_L^p}) \tag{5}$$

According to Young's equation，when contact angel θ > 0,

$$\gamma_S = \gamma_{SL} + \gamma_L \cos\theta \tag{6}$$

Combine these equations,

$$\frac{\gamma_L(1+\cos\theta)}{2\sqrt{\gamma_L^d}} = \sqrt{\gamma_S^d} + \sqrt{\gamma_S^p}\frac{\sqrt{\gamma_L^p}}{\sqrt{\gamma_L^d}} \tag{7}$$

The polar and dispersive components of the solvent is known, thus plotting the left side of Supplementary Equation 7 against $\sqrt{\gamma_L^p}/\sqrt{\gamma_L^d}$ will theoretically produce a linear line of data points. Then $\sqrt{\gamma_S^d}$ and $\sqrt{\gamma_S^p}$ can be calculated with more than two liquids with given surface tension components.

Supplementary References

1 Van Oss, C. J. *Interfacial forces in aqueous media*. second edn213, 216 (CRC press, Boca Raton, FL, 2006).

2 Wu, S. *Polymer interface and adhesion*. 151 (M. Dekker, New York, 1982).

3 Toussaint, A., Luner, P. & Mittal, K. *Contact angle, wettability and adhesion*. 385 (VSP, Utrecht, The Netherlands, 1993).

4 Schultz, J., Tsutsumi, K. & Donnet, J.-B. Surface properties of high-energy solids. *J. Colloid Interface Sci.* **59,** 277-282 (1977).

5 Sell, P.-J. & Renzow, D. Bestimmung des benetzungsverhaltens von pigmenten. *Prog. Org. Coat.* **3,** 323-348 (1975).

6 Ding, B., Kim, H., Kim, C., Khil, M. & Park, S. Morphology and crystalline phase study of electrospun TiO_2–SiO_2 nanofibres. *Nanotechnology* **14,** 532-537 (2003).

7 Samantaray, S. K. & Parida, K. Effect of phosphate ion on the textural and catalytic activity of titania–silica mixed oxide. *Applied Catalysis A: General* **220,** 9-20 (2001).

8 Parida, K. M., Samantaray, S. K. & Mishra, H. K. SO_4^{2-}/TiO_2–SiO_2 mixed oxide catalyst, I: synthesis, characterization, and acidic properties. *J. Colloid Interface Sci.* **216,** 127-133 (1999).

9 Kondo, S., Yamauchi, H., Kajiyama, Y. & Ishikawa, T. Physical and chemical properties of inactive surface hydrogen-bonded hydroxyl groups. *J. Chem. Soc., Faraday Trans. 1* **80,** 2033-2038 (1984).

10 De Gennes, P.-G., Brochard-Wyart, F. & Quéré, D. *Capillarity and wetting phenomena: drops, bubbles, pearls, waves*. 15-18 (Springer, 2003).

11 Wong, T. S. *et al.* Bioinspired self-repairing slippery surfaces with pressure-stable omniphobicity. *Nature* **477,** 443-447 (2011).
